# HIV-1 Genetic Diversity and Transmitted Drug Resistance Mutations among Patients from the North, Central and South Regions of Angola

**DOI:** 10.1371/journal.pone.0042996

**Published:** 2012-08-29

**Authors:** Joana Morais Afonso, Gonzalo Bello, Monick L. Guimarães, Marta Sojka, Mariza G. Morgado

**Affiliations:** 1 Laboratorio de AIDS & Imunologia Molecular, Instituto Oswaldo Cruz - FIOCRUZ, Rio de Janeiro, Brazil; 2 Faculdade de Medicina, Universidade Agostinho Neto, Luanda, Angola; 3 São Lucas Medical Center, Kifangondo, Angola; Centro de Biología Molecular Severo Ochoa (CSIC-UAM), Spain

## Abstract

**Background:**

Angola presents a very complex HIV-1 epidemic characterized by the co-circulation of several HIV-1 group M subtypes, intersubtype recombinants and unclassified (U) variants. The viral diversity outside the major metropolitan regions (Luanda and Cabinda) and the prevalence of transmitted drug resistance mutations (DRM) since the introduction of HAART in 2004, however, has been barely studied.

**Methods:**

One hundred and one individuals from the Central (*n* = 44), North (*n* = 35), and South (*n* = 22) regions of Angola were diagnosed as HIV-1 positive and had their blood collected between 2008 and 2010, at one of the National Referral Centers for HIV diagnosis, the Kifangondo Medical Center, located in the border between the Luanda and Bengo provinces. Angolan samples were genotyped based on phylogenetic and bootscanning analyses of the *pol (PR/RT)* gene and their drug resistance profile was analyzed.

**Results:**

Among the 101 samples analyzed, 51% clustered within a pure group M subtype, 42% were classified as intersubtype recombinants, and 7% were denoted as U. We observed an important variation in the prevalence of different HIV-1 genetic variants among country regions, with high frequency of subtype F1 in the North (20%), intersubtype recombinants in the Central (42%), and subtype C in the South (45%). Statistically significant difference in HIV-1 clade distribution was only observed in subtype C prevalence between North *vs* South (*p* = 0.0005) and Central *vs* South (*p* = 0.0012) regions. DRM to NRTI and/or NNRTI were detected in 16.3% of patients analyzed.

**Conclusions:**

These results demonstrate a heterogeneous distribution of HIV-1 genetic variants across different regions in Angola and also revealed an unexpected high frequency of DRM to RT inhibitors in patients that have reported no antiretroviral usage, which may decrease the efficiency of the standard first-line antiretroviral regimens currently used in the country.

## Introduction

HIV remains a global health problem particularly for the Sub-Saharan Africa region, where more than 22 million people live with HIV/Aids, 1.3 million adults and children are dying annually due to Aids-related diseases and is still the region most heavily affected by HIV and of major concern, especially for its contribution to the fastest moving Aids epidemic worldwide [1].

Angola, a Southwestern African country, borders Congo, the Democratic Republic of Congo (DRC), Zambia and Namibia, and is estimated to have a total population of ∼18.5 million people according to recent international reports [2]. According to the UNAIDS 2010 epidemiological fact sheets, the estimated HIV prevalence within the adult population in Angola was 2% and about 11,000 annual deaths were AIDS-related in that year [1]. This prevalence is comparable to that reported in Congo (3.2%), but much lower than those described in some African countries from the south region such as Zambia (14%), South Africa (17%), Namibia (21%) and Botswana (25%) [1]. Of note, the HIV prevalence in Angola shows a trend to decrease since 1989 when it reached 6.1% [3] and seems to be stabilizing since then [1].

Despite its relative low prevalence, the HIV/AIDS epidemic in Angola is still a cause of concern because of the high degree of HIV-1 genetic diversity observed, which posses a major challenge for prevention and treatment programs [3], [4]. The molecular epidemiology profile of the HIV-1 epidemic in Angola is characterized by the circulation of most HIV-1 group M subtypes, a high proportion of Unique Recombinant Forms (URFs), some Circulating Recombinant Forms (CRFs), and also other genetic forms that cannot be classified into the known subtypes [4]–[8]. This complex molecular pattern is similar to that described in some neighboring countries that border Angola in the North, like the DRC and Congo [9], [10].

The access of HIV-positive patients to antiretroviral therapy (ART) has substantially reduced the AIDS-related morbidity and mortality in many regions. ART access has been increasing over the last years in many African limited resource countries such as Angola, where universal access to ART was introduced by the Ministry of Health in 2004 [11] and around 11,200 HIV-infected people were being treated until 2009 [12]. The effectiveness of ART therapy, however, may be limited by the emergence of drug resistance mutations (DRM) in treated patients and by transmission of such drug-resistant viruses to drug-naïve individuals. The emergence of secondary DRM in patients failing ART in Angola has been described [7]. At the same time, the frequency of primary DRM in Angolan HIV-infected naïve patients have increased after the implementation of universal access to ART, rising from 1.6% in 2001 [13] to 5.7% in 2008–2009 [8].

So far, most comprehensive HIV molecular epidemiologic studies performed in Angola were based on samples taken 10 years ago from Luanda and Cabinda provinces, principally [3], [5], [6]. The only study on HIV diversity and primary drug resistance carried out in Angola after implementation of ART was based on the analysis of a low number (*n* = 35) of HIV-infected pregnant women attending two antenatal clinics located in Luanda [8]. Thus, limited data concerning the actual prevalence of HIV-1 subtypes and HIV primary DRM in the different country provinces is available. The aim of this work was to obtain a more update and representative picture of the HIV-1 genetic diversity and prevalence of transmitted DRM in Angola. To this end we analyze the protease and reverse transcriptase (*PR/RT*) genomic regions of 101 HIV-positive samples collected from 2008 to 2010 from patients who lived in 10 different provinces from the North, Central, and South regions of Angola.

## Materials and Methods

### Patients and Samples

Blood samples from 101 HIV-positive reported drug-naive Angolan patients were collected in three phases, from July 2008 to November 2010 (August 2008, July 2009 and November 2010) at São Lucas Medical Center (CSSL), one of the National Referral Centers for HIV diagnosis, in Kifangondo village, located in the border between Luanda and Bengo provinces, with the approval of the local Ethical Committee. The main criteria for patient inclusion in the study were recent positive diagnosis (<12 months), CD4 counts >350 cells/mm3, and disclosed to have no known exposure to antiretroviral drugs. Previous use of ARV for the prevention of Mother-to-Child HIV Transmission (pMTCT) was considered as an exclusion criterion. Patient's serological diagnosis was done with HIV 1/2 Determine rapid test (Inverness medical professional diagnostics, Florida, USA); and further confirmed by HIV-1 Western Blot (New LAV Blot I Assay, Bio-Rad, Portugal) in the Laboratory of Clinical Analysis, Faculty of Medicine, University Agostinho Neto. CD4+/CD8+ full blood count with BD FACSCalibur™ System was performed in CSSL. Whole blood and serum/plasma samples were prepared and shipped to Laboratory of Aids & Molecular Immunology, IOC/Fiocruz, Brazil for further genotyping and DRM analysis.

### Extraction, PCR amplification and sequencing of HIV-1 RNA and DNA

Viral RNA was extracted from 500 µl of HIV-1 positive plasma samples and the *pol* gene (protease, *PR* and reverse transcriptase, *RT*) was amplified by RT-PCR using the integrated ViroseqTM HIV Genotyping System kit (Abbott Molecular Inc., IL, USA), according to the manufacturer's protocol. Of samples that were negative for Viroseq, DNA was extracted from 200 µl of total blood using QIAamp viral DNA Mini Kit (QIAgen Inc., CA, U.S.A.) and amplified using a *nested* PCR method to obtain fragments of ∼1,100 bp from the *pol* (*PR/RT*) gene region as described elsewhere [14]. PCR and RT-PCR products were purified with Illustra GFX PCR DNA Kit (GE Healthcare) and sequenced using the Big Dye Terminator v3.1 cycle sequencing kit (Applied Biosystem, CA, USA). Sequencing reactions were analyzed with an ABI 3100 automated sequencer. Sequence electropherograms were visualized, inspected and assembled with Seqman v7.0 program (DNASTAR; Lasergene, Madison, Wis., USA). RNA/DNA extraction, preparation of reagents for PCR amplification and DNA sequencing carried out in three distinct areas, as well as the inclusion of negative and positive controls, were performed as part of the QA/QC protocols to avoid cross contamination and sample mix-up problems.

### Sequence alignments

Nucleotide sequences were aligned using the ClustalW algorithm implemented in MEGA v5.0 program [15]. The final alignment of around 1,100 nucleotides (nt) covered the entire *PR* and part of the *RT* region of *pol* gene (nucleotides 2252–3323 relative to HXB2). The Angolan nucleotide sequences described in the present study were aligned with representative references strains of all known HIV-1 group M subtypes (A–D, F–H, J, K), and some CRFs, particularly those that circulate in central and west-central Africa. References HIV-1 strains were retrieved from Los Alamos HIV Sequence Database (http://www.hiv.lanl.gov). Alignment is available from the authors upon request.

### HIV-1 subtype classification and recombination analyses

Three strategies were used simultaneously to classify the Angolan HIV-1 sequences as a “pure” subtype, a CRF-like, a URF, or an unclassified (U) form:

- Phylogenetic trees were built by the Neighbor-Joining (NJ) and maximum likelihood (ML) methods to verify the clustering of Angolan HIV-1 sequences with subtype and CRFs reference sequences. NJ trees were constructed under the Tamura-Nei [16] substitution model in 1000 bootstrapped data sets, as implemented in the MEGA 5.0 program. ML trees were inferred under the GTR+I+G nucleotide substitution model, selected using the jModeltest program [1], with program PhyML [2] using an online web server [3]. Heuristic tree search was performed using the SPR branch-swapping algorithm and the reliability of the obtained topology was estimated with the approximate likelihood-ratio test (aLRT) [4] based on the Shimodaira-Hasegawa-like procedure.
- Sequences were subjected to bootscanning using Simplot software version 3.5.1 [17] to confirm the subtype assignment and identify possible recombination breakpoints. Bootstrap values supporting branching with HIV-1 reference sequences were determined in NJ trees constructed using the K2-parameter substitution model [18], based on 100 resamplings, with a 250 nt sliding window moving in steps of 10 bases. Individual query sequences were compared to reference sequences from subtypes A–D, F–H, J and K.
- To further confirm the genetic structure of putative recombinant viruses, new NJ phylogenetic analyses were conducted using the fragments assigned to different subtypes according to the proposed breakpoint position(s) by the bootscanning analysis.

Complete sequences or fragments not clustering with any known HIV-1 group M subtypes or CRFs with bootstrap support of >70% were defined as U.

### Drug-Resistance Mutation Analyses

Sequences resulting from RNA and DNA HIV-1-positive Angolan samples were evaluated for HIV-1-transmitted resistance mutations as well as for hypermutation profiles. Analysis was performed according to the Calibrated Population Resistance Tool (CPR) Version 4.1 beta that uses the Surveillance Drug Resistance Mutation panel 2009 of the Stanford genotypic resistance interpretation algorithm (http://hivdb.stanford.edu/pages/links.html) [19].

### Statistical analysis

Age, gender, CD4 counts, HIV subtypes and DRM parameters were evaluated according to the regional origin of the HIV-1 positive patients included in the study. Data analyses were performed using chi-square test and considered statistically significant when *P* values were ≤0.05.

### Nucleotide sequence accession numbers

The GenBank database accession numbers for the pol sequences described in this study are JN937017 to JN937117.

## Results

### Epidemiological and clinical data

Epidemiological and clinical data describing the population enrolled in the study are presented in Table 1. Patients' median age was 35 (IQR 29–40) years old, 74.5% being women and heterosexual was the most common (96%) self-reported route of transmission. Most (76%) patients displayed CD4 T cell counts between 350–500 cells/mm3. Among the 101 HIV-1 positive Angolan patients analyzed in this study, 44 (43.6%) live in central Angola (Luanda = 22, Bengo = 16, Cuanza Norte = 6 and Cuanza Sul = 4), 35 (34.6%) in northern Angola (Cabinda = 15, Zaire = 8 and Uíge = 12) and 22 (21.8%) in southern Angola (Benguela = 10, Huíla = 3 and Namíbe = 9) since the civil war period (1992–2002), when population movements within Angola were intensified. These patients have recently moved their follow-up monitoring and testing clinic to Luanda (median date of arrival at the clinic = 2008, range between 2007 and 2010) looking for better medical assistance conditions, treatment availability and care. No statistical significant differences (*p*>0.05) were observed for age, gender or CD4 T cell counts among patients from different regions. None of the patients were under the regular antiretroviral therapy regimen, although nine patients (9%) reported being part of a spiritual group where the head prescribed some natural herbs and drugs for treatment (of which there is no information about) and six female patients (6%) later revealed (when DRM results were made available for doctors) that they were submitted to ARV (AZT+3TC+NEV or AZT+3TC+LVP/r) for preventing MTCT during delivery.

**Table 1:** Epidemiological and clinical characteristics of the Angolan HIV-infected patients from the different geographic regions.

*Parameters*	*North*	*Central*	*South*	*Total*
	*(n = 35)*	*(n = 44)*	*(n = 22)*	*(n = 101)*
Age (years)				
19–25	3 (9%)	4 (9%)	1 (5%)	8 (8%)
26–35	18 (51%)	22 (50%)	6 (27%)	46 (46%)
36–45	14 (40%)	18 (41%)	15 (68%)	47 (47%)
Gender				
Male	8 (23%)	12 (27%)	6 (27%)	26 (26%)
Female	27 (77%)	32 (73%)	16 (73%)	75 (74%)
CD4 T cell count (cells/mm3)				
≥350–500	27 (77%)	31 (70%)	19 (86%)	77 (76%)
>500	8 (23%)	13 (30%)	3 (14%)	24 (24%)

### Identification of pure HIV-1 subtypes, CRFs-like and URFs in Angolan samples

**Figure 1:**
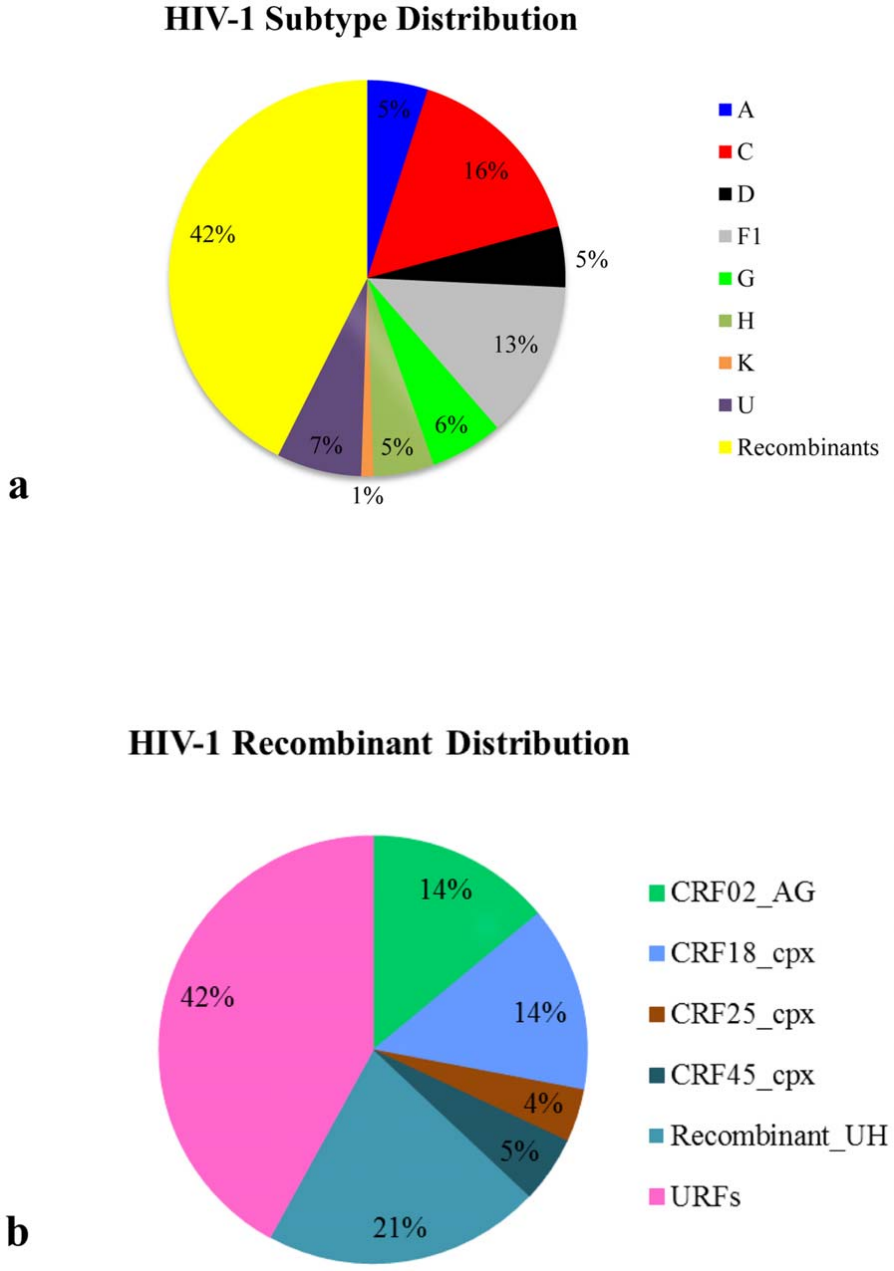
Detailed distribution of HIV-1 variants found in the Angolan population. a) Frequency of “pure” subtypes, inter-subtype recombinants and unclassified (U) sequences within the 101 HIV-1 group M Angolan samples analyzed. b) Frequency of Circulating Recombinants Forms (CRFs) and Unique Recombinant Forms (URFs) within the 42 inter-subtype recombinants samples described above.

According to the phylogenetic and bootscan analyses of the *PR/RT* region, all viruses belonged to the HIV-1 group M clade. Among the 101 samples analyzed, 51 (51%) clustered within a pure group M subtype, 43 (42%) were classified as intersubtype recombinants, and 7 (7%) were denoted as U (Fig. 1A).

**Figure 2:**
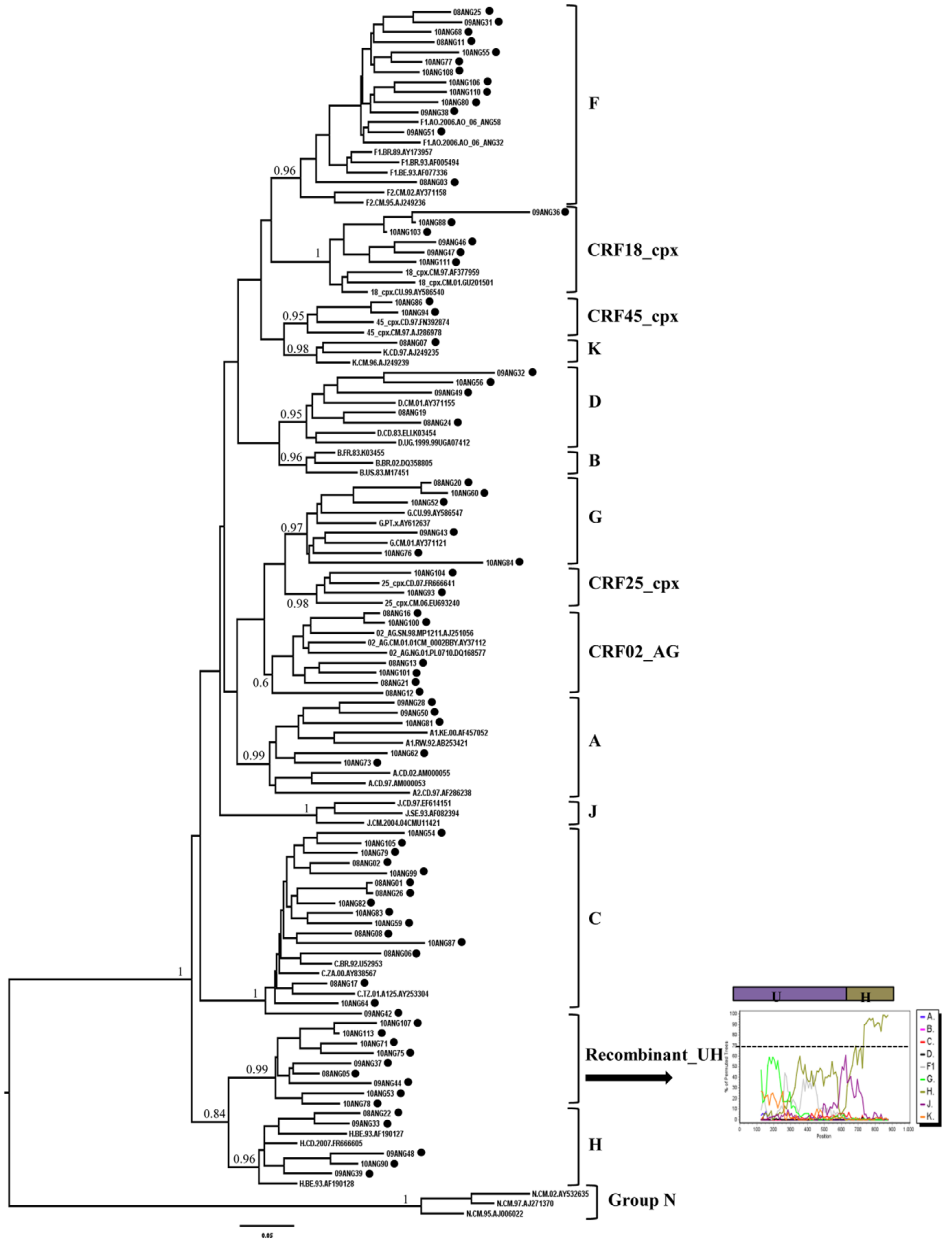
ML tree of the *pol* (PR/RT) region of Angolan viruses (black circles) classified as “pure” subtypes, CRFs-like, or the putative new recombinant_UH. Reference sequences from all HIV-1 subtypes and CRFs 02_AG, 18_cpx, 25_cpx and 45_cpx were also included. *aLRT* values are only shown at key notes. The scale represents number of substitutions per site. A representative bootscanning plot and the proposed schematic structure of recombinant_UH strains are shown. The dashed line indicates the cut-off of 70%.

**Figure 3:**
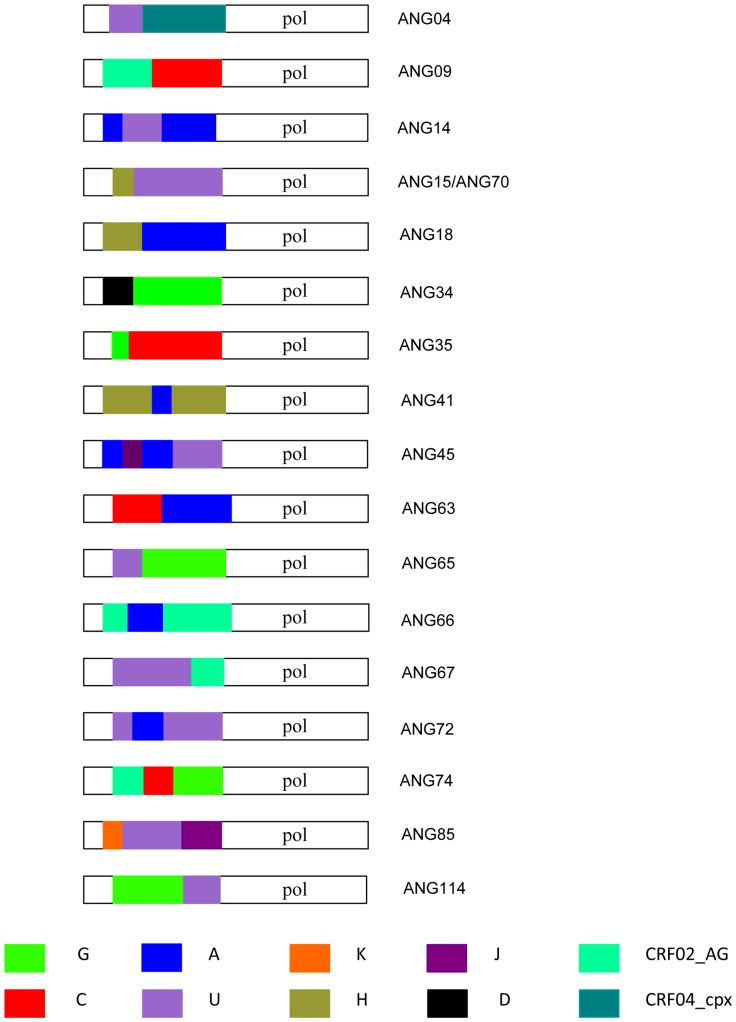
Schematic drawing showing breakpoint pattern at the *pol* (PR/RT) region of the HIV-1 URFs found in Angola. Breakpoint positions were obtained using bootscanning and phylogenetic analyses. The *pol* gene is colored according to the subtype as shown in the figure legend. The fragments not analyzed are represented in white.

**Figure 4:**
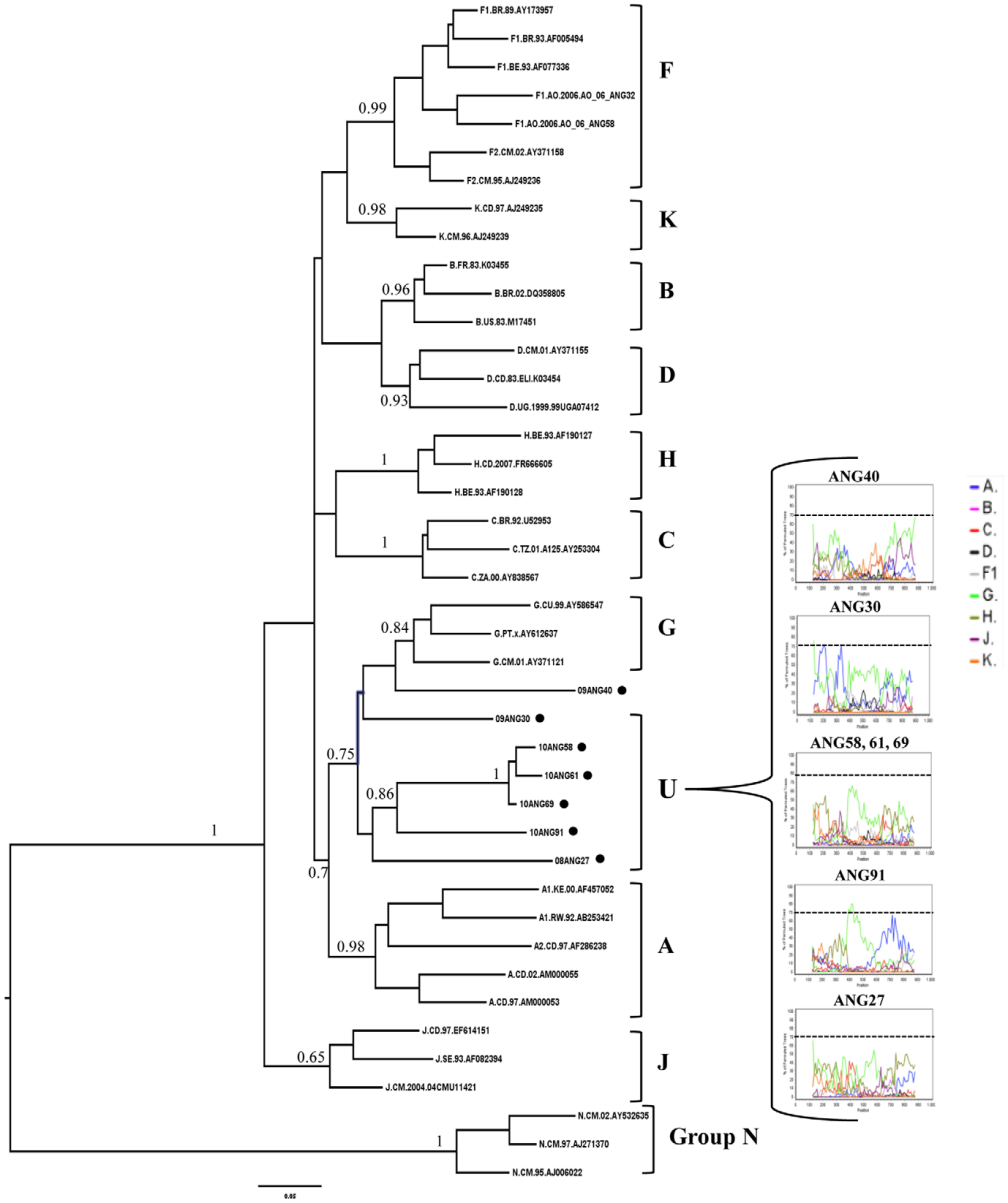
B. ML tree of the *pol* (PR/RT) region of Angolan sequences classified as U (black circles) plus reference sequences from all HIV-1 subtypes. *aLRT* values are only shown at key notes. The scale represents number of substitutions per site. Bootscanning plots of Angolan U strains are shown. Only one representative bootscanning plot is shown for the phylogenetically related Angolan U sequences ANG58, ANG61 and ANG69. The dashed line indicates the cut-off of 70%.

The most frequent pure group M subtypes verified in Angola were C (16%) and F1 (14%), followed by G (6%), A, D and H (5% each), and K (1%) (Figs. 1A and 2). Sixteen (37%) out of the 43 intersubtype recombinant sequences clustered with high support with previously recognized CRFs including: 02_AG (*n* = 6), 18_cpx (*n* = 6), 25_cpx (*n* = 2), and 45_cpx (*n* = 2) (Figs. 1B and 2). Nine (21%) recombinant sequences failed to cluster with recognized CRFs, but displayed the same UH mosaic structure at *PR/RT* region and segregated in a highly supported monophyletic cluster (Fig. 2). These sequences might be indicative of the presence of a putative new HIV-1 circulating recombinant form in Angola, although further full-length analysis will be necessary to confirm this hypothesis. So far, here on they will be denoted as recombinant_UH. The remaining 18 (42%) recombinant sequences displayed unique mosaic structures not related with any known CRF or among each other, and were classified as URFs. These URFs included recombination events between different HIV-1 subtypes (A, C, D, G, H, J and K), CRFs (CRF02_AG and CRF04_cpx) and unclassified (U) sequences (Fig. 3). Subtypes A and C, and the CRF02_AG were the recognized clades most frequently found in the mosaic genomes of the URFs. U strains were also frequently involved in recombination events, being detected in 61% of the URFs and 78% of the CRFs or “putative” CRFs (CRF02_cpx, CRF04_cpx, and recombinant_UH identified in this study. Despite the high prevalence of subtype F1 in our study population, we found no evidence of URFs or CRFs containing this subtype in their mosaic genomes. Interestingly, all seven sequences classified as “pure” U strains branched between subtypes A and G (Fig. 4), however the *aLRT* value 0.7, cannot be considered significant enough to suggest that all these clades originated from the same common ancestor. Of those seven sequences, three apparently unrelated patients (ANG58, ANG61 and ANG69) formed a highly supported (*aLRT* = 1) monophyletic cluster that was also related (*aLRT* = 0.86) with a fourth U strain (ANG91) (Fig. 4).

### Geographical distribution of HIV-1 subtypes in Angola

**Figure 5:**
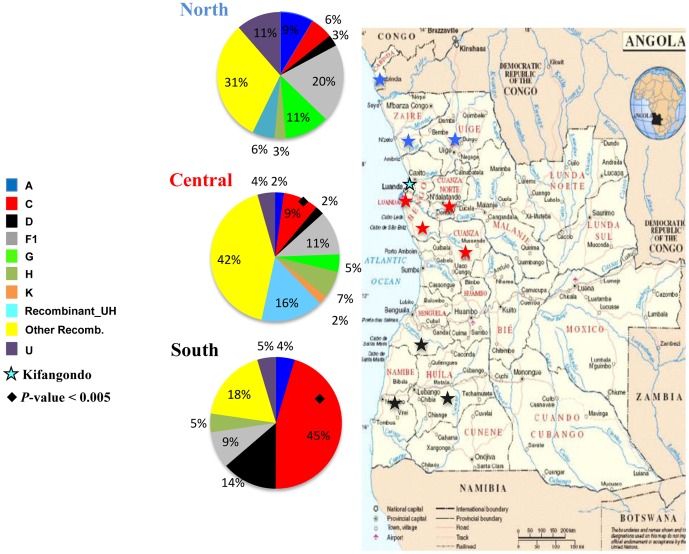
Map of Angola showing the frequency of HIV-1 genetic variants across different country regions (Central, North and South). Provinces where patients included in the present study were from are indicated: Luanda, Bengo, Cuanza Norte and Cuanza Sul provinces of the Central region (red stars); Cabinda, Zaire and Uíge provinces of the North region (blue stars); and Benguela, Huíla and Namíbe provinces of the South region (black stars). Kifangondo is indicated with a black and blue star. Statistically significant difference in HIV-1 clade distribution was observed in subtype C prevalence between North *vs* South (*p* = 0.0005) and Central *vs* South (*p* = 0.0012) regions.

The HIV-1 subtype distribution according to the region of origin of each individual demonstrates an important variation in the prevalence of different HIV-1 genetic variants among country regions (Fig. 5). The prevalence of subtype F1 decrease following a north to south axis: 20% in the North, 11% in the Central and 9% in the South. The prevalence of subtype C follows the opposite direction, increasing from north to south axis: 6% in the North, 9% in the Central and 46% in the South. The putative new recombinant_UH showed a higher prevalence in the Central region (16%), when compared to the North (6%) and South (0%) regions. A similar distribution pattern was found for the other recombinant (CRFs and URFs) whose prevalence in the Central region (42%) was higher than in the North (31%) and South (18%) regions. Other subtypes (A, D, G and H) and U strains appeared to have more uniform distributions across regions. Statistically significant difference in HIV-1 clade distribution was observed in subtype C prevalence between North *vs* South (*p* = 0.0005) and Central *vs* South (*p* = 0.0012) regions.

### Resistance to Antiretroviral Drugs

The six female patients that were submitted to ARV (3TC+AZT+NVP, or 3TC+AZT+LVP/r) for pMTCT on previous pregnancies were excluded from the analysis of transmitted DRMs, as well as those nine patients who reported being part of the spiritual group since we have no information about the drugs prescribed by the head of the group. Notably, all six female patients submitted to ARV for pMTCT displayed DRMs to RT inhibitors: two to nucleoside reverse transcriptase inhibitors (NRTI) and four to NRTI plus non-nucleoside reverse transcriptase inhibitors (NNRTI). Seven out of nine patients that reported being part of the spiritual group also presented DRMs: three displayed multiple DRM to both NRTI and NNRTI, while the other four displayed mutations to PR inhibitors (PI) only.

Among the remaining 86 HIV-infected Angolan drug-naïve patients analyzed, 14 (16.3%) individuals displayed at least one DRM (Table 2). Mutations related with resistance to NRTI were observed in 9 (10.5%) patients, M184V (9.3%) and T215F (2.3%) being the most prevalent ones. DRM to NNRTI were observed in 12 (14%) patients, K103N (9.3%) and Y181C (2.3%) being the most common ones. Seven patients (8%) displayed DRM to both classes of RT inhibitors. DRM to PI were not observed in these patients. Most patients with transmitted DRMs were between 26 and 45 years old (86%), females and males (each 50%), and displayed CD4 counts between 350–500 cells/mm3 (57%) (Table 2), following the overall trend of the study population. Distribution of DRM seems to have no association with sex, age, or subtype (*p*>0.05). Prevalence of transmitted DRMs appeared to be higher in the Central region (16%) than in North (8.6%) or South (18%) regions of Angola, although this difference was not statistically significant (*p*>0.05).

**Table 2:** Epidemiological, clinical and virological characteristics of Angolan treatment naïve patients with primary DRM.

Sample ID	Gender	CD4 (cells/mm3)	Age (years)	Subtype (PR/RT)	Geographic region	Mutations to NRTI	Mutations to NNRTI
ANG11	F	599	42	F1	North	M184V	K101E, G190A
ANG13	M	516	26	CRF02_AG	Central	-	K103N
ANG14	M	401	43	A/U/A	South	-	K103N
ANG25	F	561	20	F1	Central	M184V	K103N
ANG41	F	353	45	H/A/H	Central	-	Y181C
ANG42	M	442	30	C	South	M184V	Y181C
ANG49	M	525	31	D	South	M184V	Y106M
ANG54	F	538	35	C	South	M184V	K103N
ANG70	M	365	37	H/U	North	M184V, T215F	K103N, M230L
ANG75	F	352	28	U/H	Central	-	K103N
ANG80	M	355	39	F1	North	M184V	K103N
ANG100	F	502	19	CRF02_AG	Central	T215F, M41L	-
ANG107	M	368	34	U/H	Central	V75M, M184V	-
ANG111	F	494	29	CRF18_cpx	Central	-	K103N

NRTI, nucleoside reverse transcriptase inhibitors; NNRTI, non-nucleoside reverse transcriptase inhibitors,

## Discussion

This work represents an important update of the molecular epidemiologic scenario of the HIV/AIDS epidemic in Angola, once most previous studies were focused in analysis of samples from 2001 [4]–[6]. The phylogenetic analysis of the *pol* gene of 101 HIV-positive samples collected in Angola between 2008 and 2010 confirmed the extremely high genetic diversity of HIV epidemic characterized by the circulation of several HIV-1 group M subtypes, CRFs and URFs, as well as a high number of U strains. This complex HIV-1 molecular epidemiologic scenario is similar to that described in previous studies [4]–[8] and was probably originated by the random exportation of both pandemic and non-pandemic HIV-1 group M strains out of the epicenter in Central Africa into Angola.

The most prevalent HIV-1 clades in Angola were subtype C (16%) and subtype F1 (14%), also in agreement with some previous studies [5]–[8]. Other HIV-1 strains found at lower prevalence in our study were subtype G and CRF02_AG (6% each); subtype A, subtype D, subtype H and CRF18_cpx (5% each); CRF45_cpx and CRF25_cpx (2% each), and subtype K (1%). Of note, this is the first time that CRF18_cpx and CRF45_cpx are detected in Angola. The CRF18_cpx was identified for the first time in Cuba [20], a country that maintained social and political relationships with Angola, particularly during the civil-war; while the CRF45_cpx is a complex recombinant detected at low frequency in some Central African countries including Cameroon, Gabon, and the DRC [21].

Thirty-four HIV-1 samples from our study failed to cluster with recognized subtypes or CRFs. From these, seven samples were denoted as U and 18 samples were classified as URFs. The remaining nine sequences formed a tightly supported monophyletic clade showing identical UH mosaic structure at *pol* gene, and are candidates to be considered a new CRF. This putative new CRF, here called recombinant_UH, was the third most prevalent strain in our population (9%), behind subtypes C and F1, being detected in five of the 10 Angolan provinces analyzed. Notably, this variant is particularly prevalent in the Central region (16%), showed a lower prevalence in the North (5%), and seems to be absent in the South. This suggests that the Central region, that includes the capital Luanda, may be the epicenter of the local dissemination of this new putative CRF. Near full-length HIV genome amplification must be carried out to determine whether such U/H recombinant viruses represent a novel HIV-1 CRF previously unnoted in Angola.

The URFs represent almost 18% of sequences detected in the present study and display a complex diversity of mosaics genomes involving several subtypes (A, C, D, G, H, J, K), CRFs (02_AG and 04_cpx) and U segments. The high number of URFs circulating in Angola may imply high rates of coinfection and superinfections, which, to some extent is surprising due to Angola's low HIV-1 prevalence. We can therefore suspect that the epidemic in Angola is transmitted mainly by subpopulations of highly exposed and susceptible individuals, such as sex workers, truck drivers and within couples where men practice polygamy, a behavior very common in Angola. Alternatively, the high frequency of URFs may result from multiple introductions of unrelated recombinant strains from the neighboring countries rather than from frequent coinfection and superinfections. This could explain the paucity of local URFs containing subtype F1, one of the most prevalent variants in Angola. Due to the design and methodology used in the study, we cannot exclude the occurrence of dual/multiple infections in this population.

The circulation of HIV-1 group M strains denoted as U is an important component of the Angolan epidemic. Of note, 34 out of 101 sequences analyzed here displayed U fragments at the *pol* genomic region. For seven sequences, the complete *pol* fragment here analyzed was classified as U. Interestingly, all those seven U sequences branched between subtypes A and G indicating that all putative “pure” U clades here detected share a common ancestor with subtypes A and G. This observation, however, should be interpreted with caution because clustering of A, G and U clades was not significant (*aLRT* = 0.70). Three U sequences (ANG58, ANG61 and ANG69) formed a highly supported monophyletic cluster that was also related with a fourth U strain (ANG91) suggesting that some U viruses may have been locally disseminated in Angola. Near full-length HIV genome amplifications must be carried out to determine whether such U viruses may represent a novel HIV-1 group M subtype, a novel complex CRF or just new URFs.

Our geographic analysis revealed for the first time a non-uniform distribution of HIV-1 genetic variants across different regions in Angola. According to this analysis the South region is characterized by a high prevalence of subtype C (45.5%), the North region displays an elevated frequency of subtype F1 (20%), while the Central region exhibits a high frequency of recombinants sequences (42%), particularly of the recombinant_UH clade (16%). This heterogeneous scenario may have arisen by the frequent population movements to and from neighboring countries that happen during and after the civil war in Angola. Thus, the HIV-1 epidemic in the South region is mainly influenced by virus influx from countries located at the South and Southeast (Namibia and Zambia) that show a high prevalence of subtype C. By contrast, the more complex HIV-1 epidemic in the North and Central regions of Angola may reveals a more intense viral influx from countries located at the North and Northeast (DRC and Republic of Congo).

Our findings showed no transmitted DRM to PIs, high frequency (16.3%) of transmitted DRM to either NRTI or NNRTI, and intermediate frequency (8%) of DRM to both classes of RT inhibitors in the study population. The prevalence of transmitted DRMs appeared to be higher in Central region (16%) than North (8.6%) or South (18%) regions, reflecting perhaps the ease access to ART or the more frequent movement to other countries where treatment is available for people living in Central and South regions. Of note, a high prevalence of DRM to RT inhibitors (37%) was observed in young individuals (19–25 years), although the number of patients in that category was low (*n* = 8). A high prevalence of DRM to RT inhibitors (33%) and PI inhibitors (44%) was also observed among patients reported being part of a spiritual group where the head prescribed some natural herbs and drugs for treatment, pointing to an unrecognized use of license antiretroviral drugs in those patients. Finally, high prevalence of DRM to one or both class of RT inhibitors was also detected in a group of six female patients that were submitted to ARV for pMTCT. In Angola, two regimens for pMTCT are available: AZT+3TC+NEV, or in the absence of NEV, AZT+3TC+LVP/r. Most isolates with DRM are not fully sensitive to the standard first-line ART regimens currently used in Angola that comprises a combination of two NRTI (AZT+3TC) and a NNRTI (EFV or NEV) [11], [12], which may lead to a high rate of therapeutic failure.

The overall prevalence of transmitted DRM to RT inhibitors in our population (16.3%) is much higher than that previously estimated in Angola in 2001 (1.6%) [8]. Moreover, it is also high if we consider the WHO categorization of HIV transmitted DRM (<5% [low], 5–15% [Medium] and >15% [High]) for countries scaling-up ART [22], [23], [24] and suggests that sentinel surveillance would have to be implemented in Angola. Such temporal increase of DRM may be explained by: a) an extremely rapid dissemination of drug-resistant viruses since universal access to ART in Angola in 2004, possibly due to intermittent usage; b) the recent displacements to Angola of HIV-infected people from countries where HAART has been available for a longer period of time [25]; c) the growing unregulated and unmonitored use of antiretrovirals bought in the black market or abroad by Angolan HIV-infected patients [26]; and/or d) the non-reported use of antiretrovirals for fear of discrimination or other social issues, and difficulty of access to therapy in other regions of the country, as we observed in the present study. Of note, our estimates of transmitted DRM prevalence is also higher than that recently described for a group of pregnant women diagnosed in Luanda between 2008 and 2009 (5.7%) [13]. More comprehensive studies are necessary to confirm the actual prevalence of primary DRM in Angola.

In conclusion, this study suggests that the HIV-1 epidemic in Angola has been probably shaped by both frequent population movements to and from neighboring countries related to the political instability observed in Angola since the early 1960s, and by the local dissemination of some specific HIV variants. This study also revealed an unexpected high frequency of DRM to RT inhibitors in patients that have reported no antiretroviral usage before being included in the study. This may uncover frequent transmission of drug-resistant virus to newly HIV-infected individuals and/or common unmonitored use of antiretrovirals drugs among Angolan HIV-infected patients. Both phenomena could seriously decrease the efficiency of the standard first-line antiretroviral regimens currently used in the country and certainly deserve further attention.
